# Influence of bacteria on the maintenance of a yeast during *Drosophila melanogaster* metamorphosis

**DOI:** 10.1186/s42523-021-00133-0

**Published:** 2021-10-03

**Authors:** Robin Guilhot, Antoine Rombaut, Anne Xuéreb, Kate Howell, Simon Fellous

**Affiliations:** 1 CBGP, INRAE, CIRAD, IRD, Montpellier SupAgro, Univ Montpellier, Montpellier, France; 2 Faculty of Veterinary and Agricultural Sciences, University of Melbourne, Parkville, VIC 3010 Australia

**Keywords:** Maintenance through metamorphosis, Symbiosis, Microbial interactions, *Drosophila melanogaster*, *Saccharomyces cerevisiae*, Extracellular bacteria

## Abstract

Interactions between microorganisms associated with metazoan hosts are emerging as key features of symbiotic systems. Little is known about the role of such interactions on the maintenance of host-microorganism association throughout the host’s life cycle. We studied the influence of extracellular bacteria on the maintenance of a wild isolate of the yeast *Saccharomyces cerevisiae* through metamorphosis of the fly *Drosophila melanogaster* reared in fruit. Yeasts maintained through metamorphosis only when larvae were associated with extracellular bacteria isolated from *D. melanogaster* faeces. One of these isolates, an Enterobacteriaceae, favoured yeast maintenance during metamorphosis. Such bacterial influence on host-yeast association may have consequences for the ecology and evolution of insect-yeast-bacteria symbioses in the wild.

**Supplementary Information:**

The online version contains supplementary material available at 10.1186/s42523-021-00133-0.

## Background

In microorganism-metazoan associations, the microorganism can influence host phenotype while the host can affect microbial multiplication and dispersal [1]. Such interactions may be influenced by interactions between microorganisms themselves, a phenomenon that is generally better understood in the context of parasitism than in the context of beneficial symbiosis [2, 3]. Beneficial microorganisms can however interact in a wide variety of mechanisms [4–6] that affect each of them as well as the phenotype of their host [7–11].

Fungal-bacterial interactions have important implications for human health, agricultural productivity, and food production [12–15]. In non-cultivated systems, yeasts and other fungi associate with extracellular bacteria, including in decaying plant materials where they interact with the larvae and adults of saprophagous insects such as *Drosophila* flies. Symbioses between *Drosophila* and either yeasts or bacteria have been extensively studied separately. However, both yeasts and bacteria may affect *Drosophila* physiology, nutrition, reproduction, and behavior [16–22] and may maintain through the *Drosophila* life cycle [23–26]. The handful of studies considering both yeast and bacteria shows that interactions between these microorganisms can modulate fly behavior [27] and nutrition [28] and that bacteria can affect fly attraction to yeast [29]. Yet, there is little information on how such interactions between yeasts and bacteria may affect their transmission from the host or their maintenance among host life stages, which would have consequences for the ecological dynamics and symbiotic relationships of flies with microorganisms.

It is established that *Drosophila* flies influence bacteria and yeasts life cycles through effects on local multiplication and dispersal [24, 30–34]. For example, adult flies, attracted by bacteria- or yeast-emitted volatiles, may acquire a microorganism (e.g. through feeding) and eventually deposit it in a fruit where larvae develop. In addition, bacteria and yeasts that interact with larvae may survive insect metamorphosis, a phenomenon that may allow their acquisition by freshly emerged adults and subsequently their dispersal to new resource sites. The maintenance of yeasts or bacteria throughout the *Drosophila* life cycle has been investigated [26, 35], including their maintenance from larvae to adults through metamorphosis (also known as transstadial maintenance or transstadial transmission) [23, 24, 36, 37]. However, it is not known whether interactions between such microorganisms affect microbial maintenance through *Drosophila* life stages. To explore this question, we investigated the maintenance of a wild yeast isolate during the metamorphosis of *Drosophila melanogaster* larvae that have been associated to different extracellular bacteria. We hypothesized that the identity of bacteria associated with larvae would affect yeast maintenance through fly metamorphosis. We found that the presence of the yeast in adults only occurred when larvae were associated with bacteria and that yeast frequency depended indeed on the identity of these bacteria.

## Methods

### Biological material

We used a *Drosophila melanogaster* (Meigen, 1830) Oregon-R strain usually maintained on a banana-based diet (233 g L^−1^ banana, 62 g L^−1^ sugar, 62 g L^−1^ dead yeast, 25 g L^−1^ ethanol, 10 g L^−1^ agar and 5 g L^−1^ nipagin) at 21 °C with a 14 h/10 h day-night cycle.

The bacterial strains used had been isolated from feces of adult flies from the colony presented above [38]. The bacterial strains were identified as *Staphylococcus* sp. (accession number MK461976 in the NCBI database), *Enterococcus* sp. (MK461977), an Enterobacteriaceae (MK461978) and an Actinobacteria (MK461979). While these bacterial strains do not belong to lactic acid and acetic acid bacteria groups that usually dominate the *Drosophila* gut microbiota, bacterial taxa close to them have frequently been identified as associated with laboratory and wild populations of *Drosophila melanogaster* [39–42]. Moreover, microbial species and strains can evolve rapidly with large consequences on their effects on host phenotype [43, 44]. To understand symbiosis, low taxonomical resolution may be partly offset by the study of microbial effects on host phenotype in relevant experimental conditions, as this was done for the bacterial strains used in the present study [38].

A strain of the yeast *Saccharomyces cerevisiae* (Meyen ex Hansen, 1883) was isolated from a wild Drosophilid captured in the *‘Le Domaine de l’Hortus’* vineyard, near Montpellier in southern France. The yeast was isolated by the fly walking across the surface of an agar plate and purified before DNA being extracted (methods described in Lam and Howell 2015 [45]). The yeast was identified using PCR amplification of the 26S ribosomal DNA region using the NL1 and NL4 primers [46]. *S. cerevisiae* have been previously detected in several natural *Drosophila* populations [32, 47, 48]; Hoang et al. (2015) have argued other yeast species frequently associated with flies should be studied as well.

### Experimental design

The experiment was conducted in sterile tubes at 24 °C. Each tube contained twenty *D. melanogaster* eggs manually deposited on a small wound of a surface-sterilized grape berry that was inoculated, or not, with specific microorganisms.

Before use, grape berries were dipped in a 2% bleach solution and rinsed with sterile water to remove all microorganisms present at the surface of the fruit. Fruit skin was then slightly incised to create an artificial wound to deposit fly eggs and specific microorganisms. *Drosophila* eggs were gently collected from grape juice plates exposed to groups of conventionally reared *D. melanogaster* Oregon-R females for 12 h. Such plates were supplemented with the antibiotic streptomycin (1 mg L^−1^, from a standard streptomycin solution of 1 mg mL^−1^ in 1 mM EDTA (Sigma-Aldrich ref. 85886)) to inhibit growth of female-associated bacteria. Repeated assays (plating on Lysogeny Broth (LB) solid media incubated at 24 °C) showed that both berries and eggs were free of cultivable bacteria and yeasts.

After egg deposition on fruit incision, fruit flesh was inoculated with 10^4^ *S. cerevisiae* yeast cells (suspended in 10 µl of sterile phosphate-buffered saline solution (PBS), from overnight culture in LB liquid media at 24 °C). Fruit incisions were then inoculated, or not, with the different bacteria (following the procedure described above). Experimental treatments were: no bacteria (‘Control’, i.e. PBS without bacterial cells; n = 18 rep.); one of the four bacterial strains (10^4^ cells; n ‘*Staphylococcus*’ = 13 rep., n ‘*Enterococcus*’ = 13 rep., n ‘Enterobacteriaceae’ = 11 rep., n ‘Actinobacteria’ = 13 rep.); and a mixture of the four bacteria (2.5 × 10^3^ cells of each bacteria; n = 9 rep.). Replicates were organized in eleven blocks launched over four days.

Newly formed *Drosophila* pupae were removed daily from their tube with a sterile fin brush and placed in a new sterile tube until adult emergence. This procedure reproduces natural insect behavior as most *D. melanogaster* larvae usually crawl out of their substrate before pupation [49, 50], which incidentally prevent the exposure of most young adults to the microorganisms present in the larval substrate. As it was not logistically feasible to sample every adult independently, we randomly selected a single pupa for each grape berry and pooled all the adults, females and males, that emerged the same day than this pupa. To detect yeast and bacterial cells in freshly emerged adults, sampled adults were homogenized in sterile PBS with two Ø3 mm glass balls using a Tissue Lyser II (Qiagen). Serially diluted fly samples were then plated on LB plates. Exact location of the microbial cells detected in the samples, i.e. either inside the fly, at the surface or both, remains therefore unknown. After incubation for 48 h at 24 °C, colonies of the five microorganisms (yeast and bacteria) were distinguished according to their morphology (shape, color, transparency, and texture) as described in a previous study [38]. This method gave robust results as (i) preliminary essays confirmed growth of each microorganism on LB plates incubated for 48 h at 24 °C, (ii) repeated molecular essays (PCR amplification and Sanger sequencing from pure colonies) attested specificity of each microorganism’ morphology under our experimental conditions, and (iii) no microbial growth was detected in fruit and insect samples from experimental controls (fruits without flies or fruits with yeast- and bacteria-free flies).

As microbial content of fly adults may be partly linked to microbial growth in the larval fruit, we collected the remaining juice from grape berries two days after the formation of the last pupa. Serially diluted fruit samples were plated on LB plates to detect yeast and bacterial cells as described above.

In parallel to the experiment on grape berries, bacteria were inoculated on cubes of laboratory banana-based diet (containing lysed yeast extract) following the procedure above to assess bacteria transstadial maintenance in their environment of origin, being the nutritive medium used to rear the fly colony. The six different bacterial treatments were: no bacteria (‘Control’, i.e. PBS without bacterial cells; n = 12 rep.); one of the four bacterial strains described above (n ‘*Staphylococcus*’ = 12 rep., n ‘*Enterococcus*’ = 7 rep., n ‘Enterobacteriaceae’ = 8 rep., n ‘Actinobacteria’ = 11 rep.); and a mixture of the four bacteria (n = 14 rep.). Replicates were organized in fifteen blocks launched over four days.

### Statistical analyses

We tested whether larval bacteria would influence yeast transstadial maintenance. We estimated yeast transstadial maintenance in groups of 1 to 11 freshly emerged adults (median = 5, IQR = 4). Yeast-positive samples contained 1 to 150 cells per adult fly (Additional file 1: Fig. S1). The variation in number of cells was not investigated statistically due to low statistical power. Whether live yeast cells were present or not was analyzed using a generalized linear model with binomial distribution and logit link function. Tested factors comprised bacterial treatment, number of adults in the groups, yeast concentration in the fruit, age of the adults, and experimental block. Backward model selection allowed to eliminate non-significant terms (yeast concentration in the fruit and age of the pooled flies) from the initial complete model. Post hoc contrasts were used to detect significant differences between bacterial treatment levels. Numbers of replicates varied among bacterial treatments due to differential larval mortality. However, the analysis of larval survival revealed no significant effect of the bacteria on this trait [38]. Low statistical power did not enable testing the interaction between bacterial treatment and number of adults in the groups (but see Additional file 1: Fig. S2 for a presentation of these results). Our biological material (i.e. wild and laboratory strains and populations) informs on the factors that can influence transstadial symbiont maintenance in a qualitative fashion and does not indicate their quantitative occurrence in the field.

To test the effect of the bacterial treatment on the yeast concentration (log-transformed) in larval fruit substrate, we used a linear mixed model with Restricted Maximum Estimate Likelihood. Experimental block was defined as a random factor.

All analyzes were performed using JMP (SAS, 14.1).

## Results

**Fig. 1:**
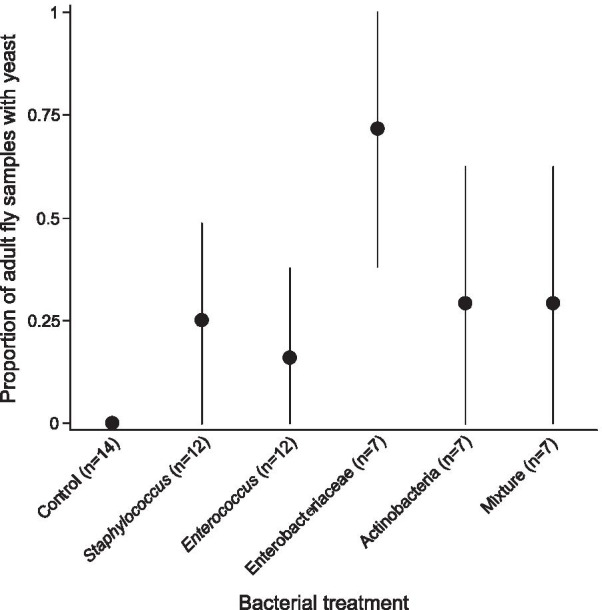
Transstadial maintenance of *Saccharomyces cerevisiae* in response to bacterial treatment. Symbols indicate the proportion of groups of freshly emerged adult flies containing yeasts per bacterial treatment (n = number of adult groups per bacterial treatment). The 95% binomial confidence intervals were calculated using normal approximation method. These results are qualitative as we used groups of adult flies to estimate yeast transstadial maintenance (Additional file 1: Fig S2)

Bacterial treatment significantly affected *S. cerevisiae* presence in freshly emerged adult flies (χ^2^ = 20.30, df = 5, p = 0.001). Yeasts were not detected in adult flies that emerged from control treatments, unlike treatments with bacteria at the larval stage (contrast ‘All treatments with bacteria’ vs ‘Control’: χ^2^ = 11.2, df = 1, p < 0.0001). Young adult flies that developed associated with the Enterobacteriaceae alone or in mixture with the other bacteria were more likely to harbor live yeast cells than the other treatments with bacteria at the larval stage (contrast ‘With Enterobacteriaceae’ vs ‘All other treatments with bacteria’: χ^2^ = 4.52, df = 1, p = 0.03) (Fig. 1). The number of individuals in the assayed group significantly and positively affected the likelihood of yeast observation (χ^2^ = 7.54, df = 1, p = 0.01) (Additional file 1: Fig. S2) – supporting the need to include this factor in all the analyses. The age of freshly emerged adult flies (χ^2^ = 0.65, df = 1, p = 0.42) and the yeast concentration in the larval medium (χ^2^ ~ 0, df = 1, p ~ 1) did not significantly influence yeast presence in adults.

**Fig. 2:**
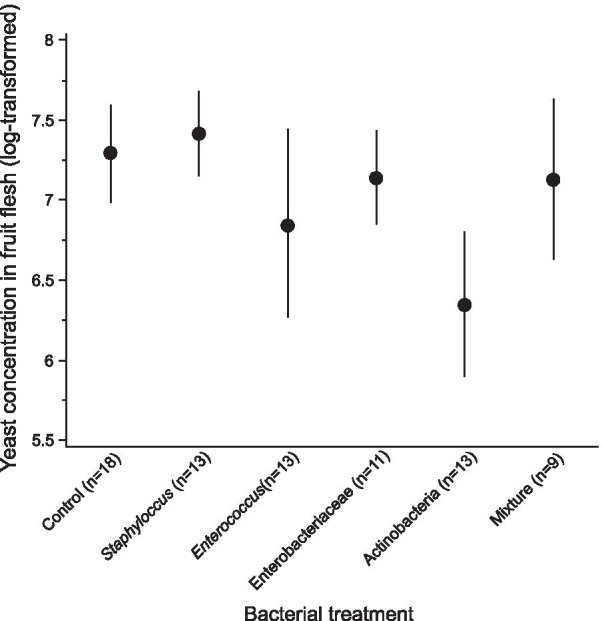
Yeast concentration in grape berry flesh after the formation of the last pupa. Concentration is expressed in number of yeast cells per 200 µl of fruit flesh. Symbols indicate mean ± standard error of the mean (SEM)

Bacterial treatment did not significantly affect yeast concentration in the medium two days after the formation of the last pupa (F_5,49_ = 1.18, p = 0.33) (Fig. 2). Yeast presence in fruit flesh was detected in all replicates but one.

Bacteria could be observed in young adults that emerged from most combinations of larval environment (i.e. grape berry and laboratory medium) and bacterial treatment (Additional file 1: Fig. S3). When bacteria were detected, load varied from 1 to 33 bacterial cells per adult fly, a variation that was not investigated statistically due to low statistical power. The observation of the inoculated bacteria in emerged adults suggest that such bacteria sampled in laboratory adults reared on artificial diet could associate with larvae, even in fruit substrate.

## Discussion

We studied the influence of bacteria on the maintenance of the yeast *Saccharomyces cerevisiae* throughout fly metamorphosis. Our results show that larval bacteria influenced transstadial maintenance of yeasts (Fig. 1). In control treatments where no bacteria were inoculated, yeasts were not found in freshly emerged adult flies. On the contrary, the presence of bacteria at the larval stage favored yeast maintenance through host metamorphosis. In particular, inoculation by our Enterobacteriaceae isolate (alone or in mixture) led to greater *S. cerevisiae* transstadial maintenance than observed when other bacteria were inoculated (Fig. 1). The propensity to increase yeast maintenance hence seemed to vary among bacteria.

It is well known that coinfecting symbionts (mutualistic as parasitic) often affect each other’s horizontal transmission to new hosts in holometabolous insects [51–54] and other multicellular organisms [3, 51, 55–57]. We know a single other case of microbial interactions affecting symbiont maintenance throughout complete metamorphosis: in *Galleria mellonella* butterflies, the bacterium *Enterococcus mundtii* interacts with host immunity during the pupal stage to shape adult bacterial microbiota [58]. Our experiment shows bacteria can affect yeast transstadial maintenance in *D. melanogaster*. The experimental design of the present study however prevents drawing excessive conclusions regarding the magnitude of the phenomenon *in natura*. This would have necessitated the testing of a greater number of microbial strains in various host genotypes, including isolates recently isolated from the field or more frequently found in wild *Drosophila* flies [25]. Follow-up studies are therefore necessary to quantify the weight of interactions between microbial symbionts on their transstadial maintenance in the field.

What mechanisms may underlie microbial transstadial maintenance, and how do bacteria may affect this process? The maintenance of *S. cerevisiae* yeasts and several bacterial strains throughout *Drosophila* metamorphosis are congruent with previous reports of the transstadial maintenance of extracellular microorganisms in Drosophilids [23, 24, 36, 59], other Dipterans [53, 60–66] and other holometabolous insects [67]. During metamorphosis, microorganisms could maintain on either the inner or outer walls of the pupal chamber [68, 69]. In *D. melanogaster*, bacterial cells of *Escherichia coli* were found associated with the internal pupal membrane [23]. Alternatively, young adults might associate with microorganisms by consuming their own meconium—the remaining of larval midgut that is excreted after adult emergence [21, 53, 70]. The mechanism of bacterial influence on yeast maintenance through metamorphosis is not clear either. The Enterobacteriaceae isolate that increased yeast maintenance, despite presenting a wide metabolic spectrum [38], is unlikely to have improved fruit quality by concentrating or synthetizing nutrients [71] as there was no significant effect on fly phenotype in this context of development in fruit [38]. The concentration of yeast cells in fruit did not correlate with the presence of yeasts in the freshly emerged adults and was not affected by the bacterial treatment (Fig. 2). This lack of quantitative relationships suggests that the maintenance of yeasts through metamorphosis may be determined by qualitative processes, involving host or yeast physiology, rather than mere cell numbers. Several bacteria are known to interact with *Drosophila* host signaling (e.g. [18, 19]). Symbiotic bacteria could therefore elicit host or yeast physiological responses in a way that would affect the likelihood of transstadial maintenance.

In the wild, such yeast transstadial maintenance in *D. melanogaster* may have consequences for the spatial spread of the yeast and the evolution of the fly-yeast association*.* Yeasts would benefit association with insects to disperse among the ephemeral patch of resources formed by fruits [72]. *Drosophila* adults could contribute to yeast dispersal through two mechanisms. Firstly, it is established that yeasts produce chemical volatiles that attract adult flies [17, 34, 73–77], which favors their acquisition and vectoring by insects to new resource patches [34]. Whether this phenomenon reflects yeast adaptation to insect vectoring is debatable [78]. Secondly, yeast maintenance through *Drosophila* metamorphosis—as demonstrated here—would enable the dispersal to new resource patches of larval symbionts (e.g. fruit, possibly infested with insect larvae) by colonized emerging adults. Such continuity in symbiosis over the life cycle would select larval symbionts for beneficial effects on host fitness [79]. The microbial strains the most beneficial to larval development (for example in terms of larval survival) would be the best dispersed to new resources patches by favoring the development of vigorous or numerous adult hosts. Furthermore, the maintenance of larval microbial symbionts until adult emergence may also benefit the host as freshly emerged adults could be less susceptible to opportunistic pathogens due to symbiont prior presence [58, 80, 81]. As transstadial maintenance of larval symbionts could have implication for the dynamics and evolution of both hosts and microorganisms, the influence of bacteria on yeast maintenance our results suggest in this study therefore illustrates new and unanticipated consequences of bacterial association with insects.

## Conclusions

Microbial interactions are emerging as key features of symbiotic systems [1, 82, 83], including associations between microorganisms and *Drosophila* flies [10, 27]. Our results suggest that a bacterial member of the *Drosophila* microbiome can influence the maintenance of a yeast through the insect metamorphosis. Such phenomenon may have consequences for the ecology and evolution of insect-yeast-bacteria symbioses in the wild. Although studying microbial symbionts in isolation may be attractive experimentally, our results illustrate that understanding the nature and diversity of host-microorganism relationships necessitates encompassing the complexity of natural communities.

## Supplementary Information

**Additional file 1:** **Figures S1, S2 and S3**. **Fig. S1:** Number of yeast cells per freshly emerged adult fly; **Fig. S2:** Relationship between the number of young adult flies in the groups and the likelihood of yeast transstadial maintenance; **Fig. S3:** Transstadial maintenance of bacteria in grape berries and in laboratory medium.

## Data Availability

The dataset is available in the open data repository Zenodo (https://doi.org/10.5281/zenodo.5069807).

## Abbreviations

rDNA: Ribosomal deoxyribonucleic acid
EDTA: Ethylenediaminetetraacetic acid
LB: Lysogeny broth
PBS: Phosphate-buffered saline solution
PCR: Polymerase chain reaction
SEM: Standard error of the mean
