# Supplementary material for: Influence of bacteria on the maintenance of a yeast during Drosophila melanogaster metamorphosis
Source: Anim Microbiome. 2021 Oct 3;3:68. doi: 10.1186/s42523-021-00133-0 (PMC8489055; doi:10.1186/s42523-021-00133-0)

Supplementary Material

Influence of bacteria on the maintenance of a yeast during *Drosophila melanogaster* metamorphosis

Robin Guilhot[1], Antoine Rombaut[1], Anne Xuéreb[1], Kate Howell[2], Simon Fellous[1]

[1]CBGP, INRAE, CIRAD, IRD, Montpellier SupAgro, Univ Montpellier, Montpellier, France
[2]Faculty of Veterinary and Agricultural Sciences, University of Melbourne, Parkville, Vic 3010, Australia

Fig. S1. Number of yeast cells per freshly emerged adult fly (log-transformed). Filled dots indicate mean ± SEM and open dots indicate individual values.

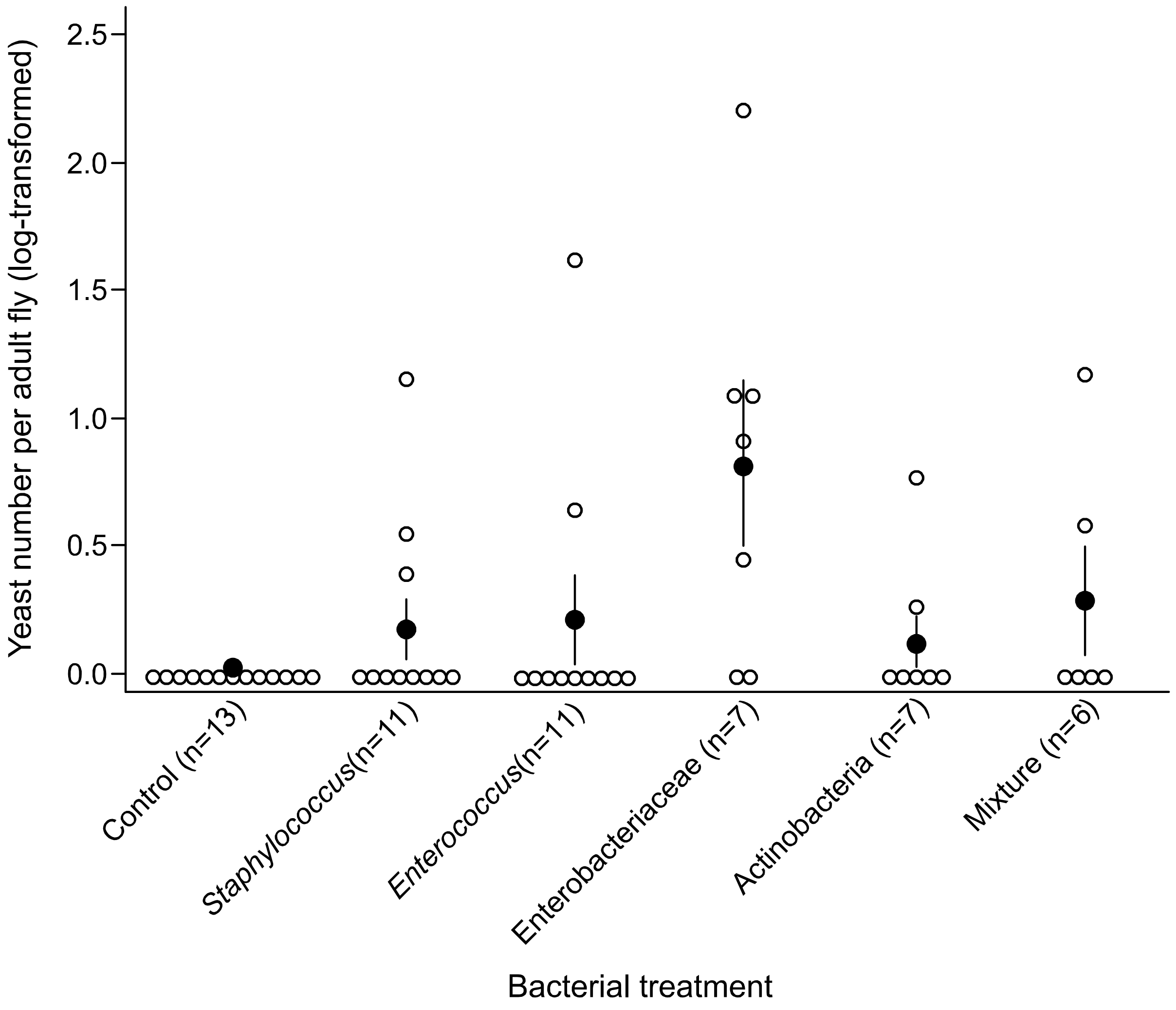

Fig. S2. Relationship between the number of young adult flies in the groups and the likelihood of yeast transstadial maintenance. The number of freshly emerged adult flies in the groups significantly and positively affected yeast detection ($\chi^2 = 7.54$, df = 1, $p = 0.01$). Filled dots indicate the proportion of adult groups containing yeast per number of adult flies in the groups (all treatments together). 95% binomial confidence intervals were calculated using normal approximation method. Other symbols indicate the proportion of adult groups containing yeast per number of adult flies in the groups for each bacterial treatment.

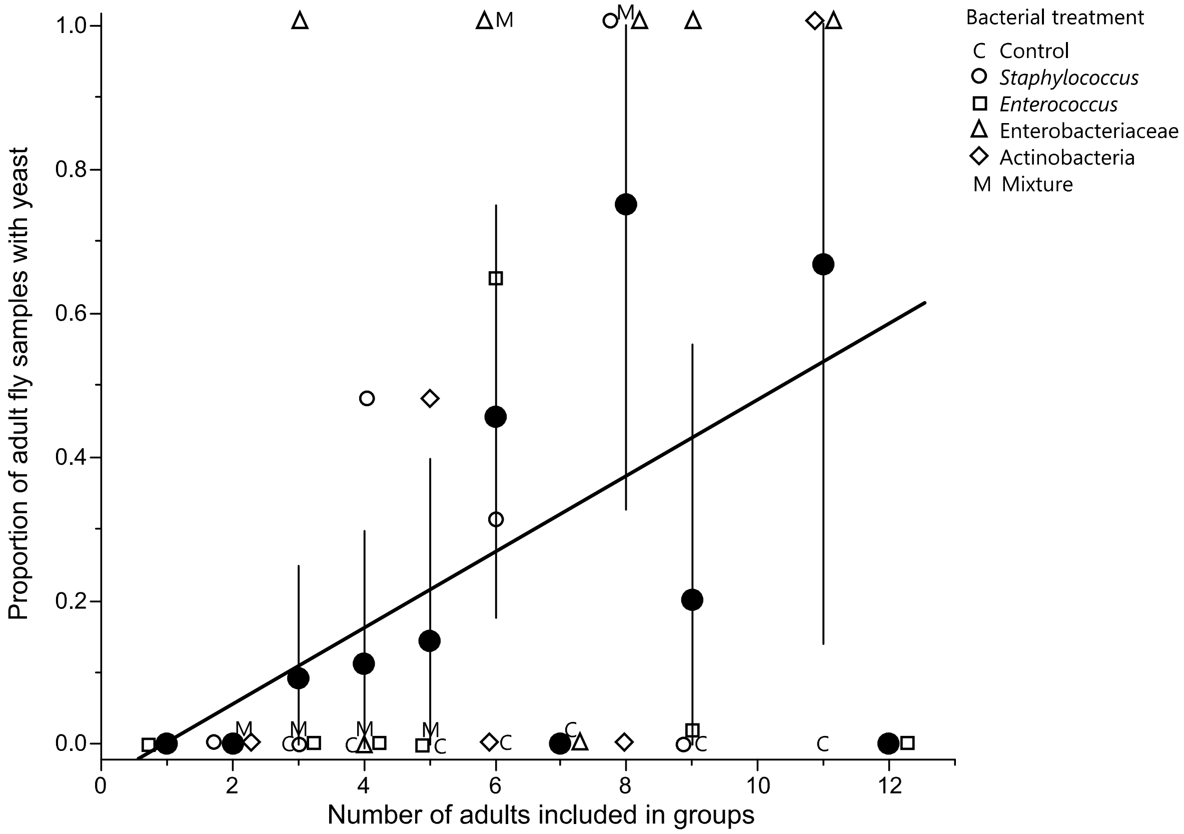

Fig. S3. Transstadial maintenance of bacteria in grape berries (A) and in laboratory medium (B). Symbols indicate the proportion of groups of freshly emerged adults containing bacteria for each bacterial treatment (n = number of groups). 95% binomial confidence intervals were calculated using normal approximation method.

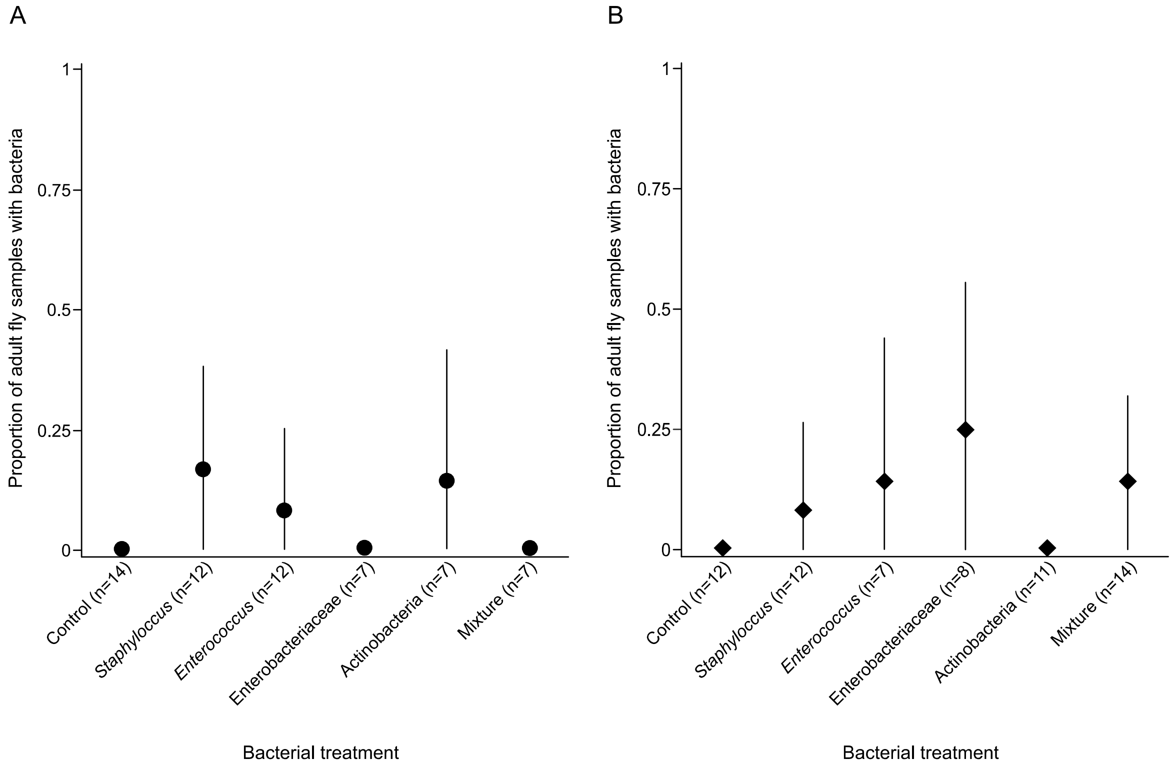

Supplement: Supplementary file 1 — Additional file 1: Figures S1, S2 and S3. Fig. S1: Number of yeast cells per freshly emerged adult fly; Fig. S2: Relationship between the number of young adult flies in the groups and the likelihood of yeast transstadial maintenance; Fig. S3: Transstadial maintenance of bacteria in grape berries and in laboratory medium. [file 42523_2021_133_MOESM1_ESM.pdf]
